# Supplementary figures and images for: Bioinformatics analysis of genes associated with disulfidptosis in spinal cord injury
Source: PLoS One. 2025 Feb 14;20(2):e0318016. doi: 10.1371/journal.pone.0318016 (PMC11828381; doi:10.1371/journal.pone.0318016)

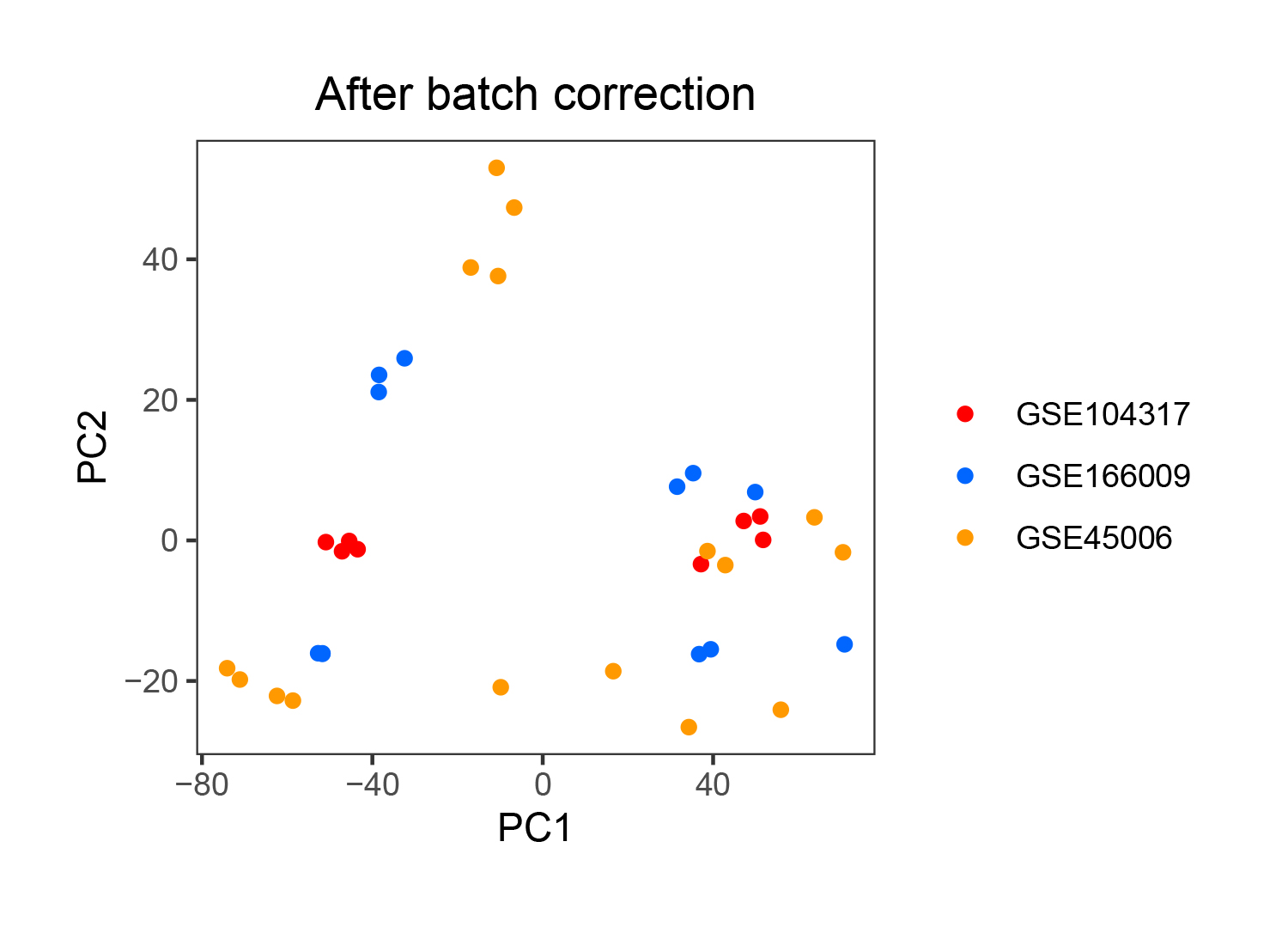

Supplement: S1 Fig — (JPG) [file pone.0318016.s001.jpg]
